# Supplementary material for: Geo-Spatial Characteristics of 567 Places of Tick-Borne Encephalitis Infection in Southern Germany, 2018–2020
Source: Microorganisms. 2022 Mar 17;10(3):643. doi: 10.3390/microorganisms10030643 (PMC8953713; doi:10.3390/microorganisms10030643)
Supplement: Supplementary file 1 [file microorganisms-10-00643-s001.zip › microorganisms-1638995-supplementary.pdf]

Article

Geo-Spatial Characteristics of 567 Places of Tick-Borne Encephalitis Infection in Southern Germany, 2018–2020

Amelie M. Friedsam, Oliver J. Brady, Antonia Pilic, Gerhard Dobler, Wiebke Hellenbrand and Teresa M. Nygren

Supplementary Materials

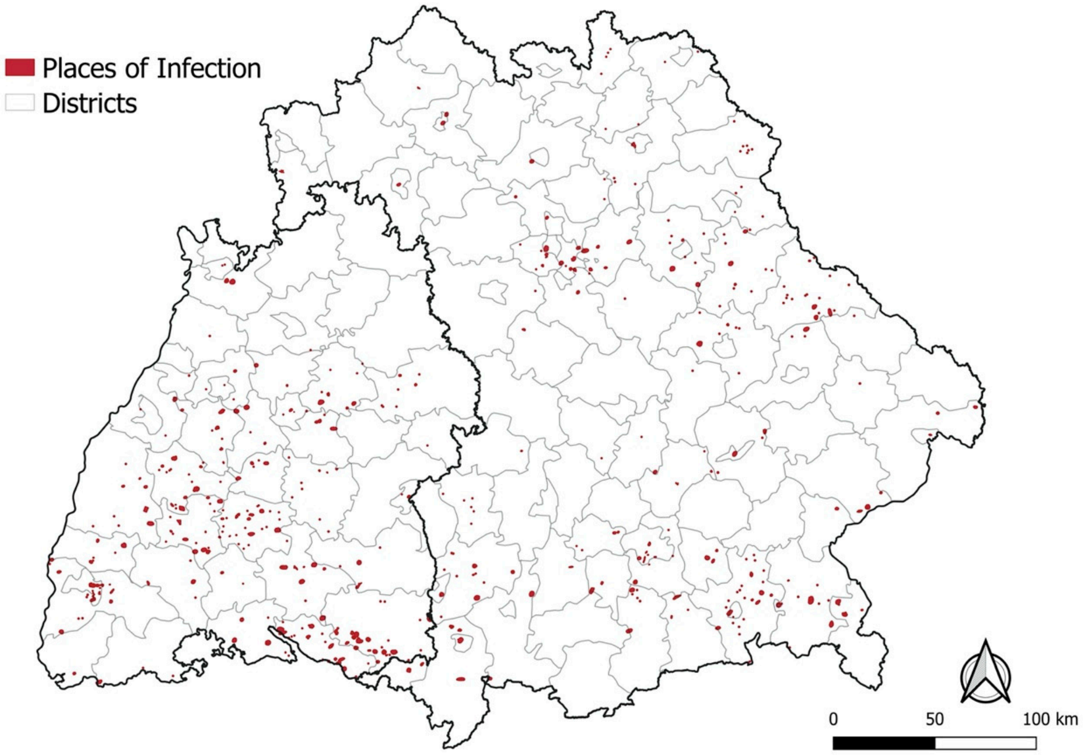

Figure S1. Self-reported places of infection (n = 567) by 359 notified TBE cases in the study region Bavaria and Baden-Wuerttemberg, 2018–2020.

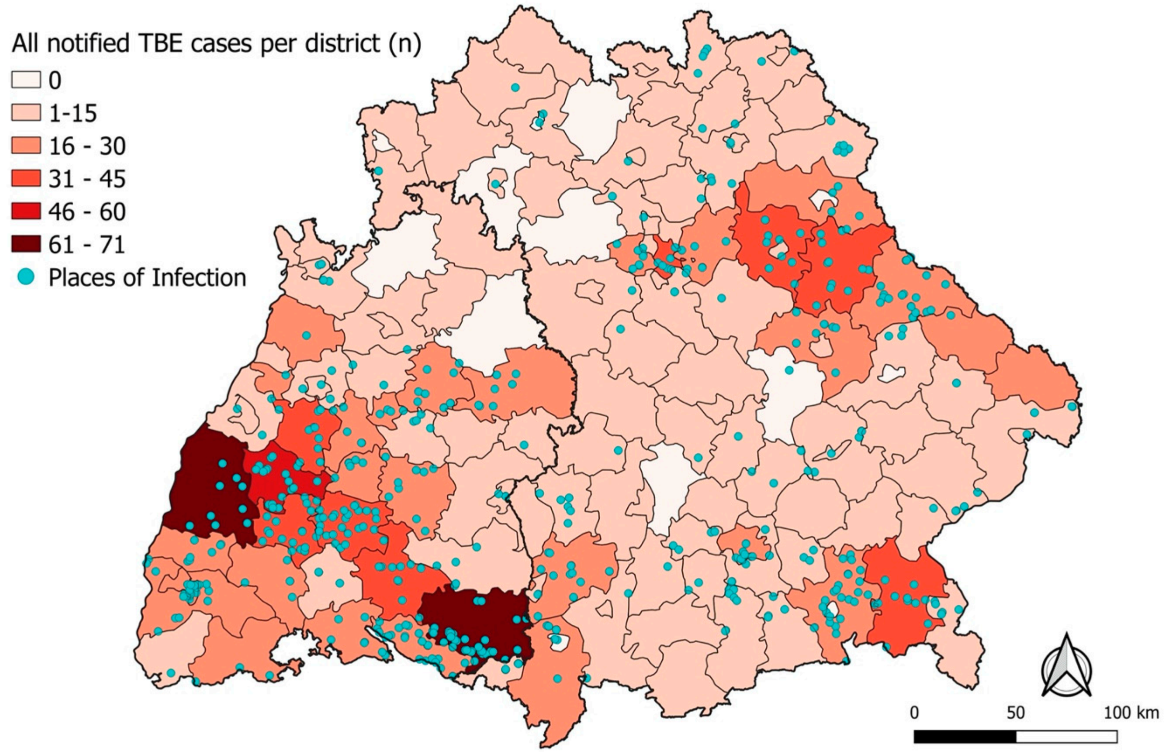

Figure S2. Spatial representativeness of the study data; Self-reported places of TBE infection compared with overall notified TBE cases per district; the choropleth map is colour-coded by the absolute count of all notified TBE cases per district between 2018 and 2020.

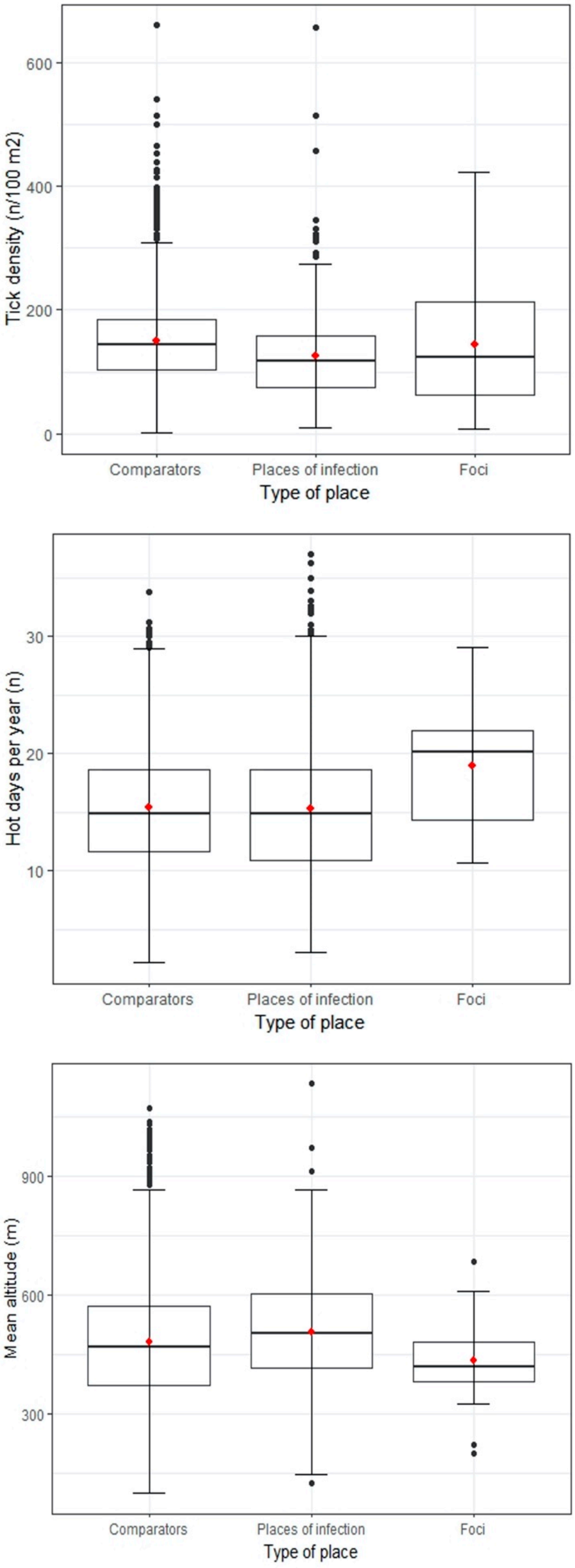

Figure S3. Distribution of tick density (*n*/100 m^2), hot days (>30 °C) per year (*n*), and altitude for comparator places (*n* = 1701), places of TBE infection (*n* = 567), and natural TBE foci (*n* = 41).

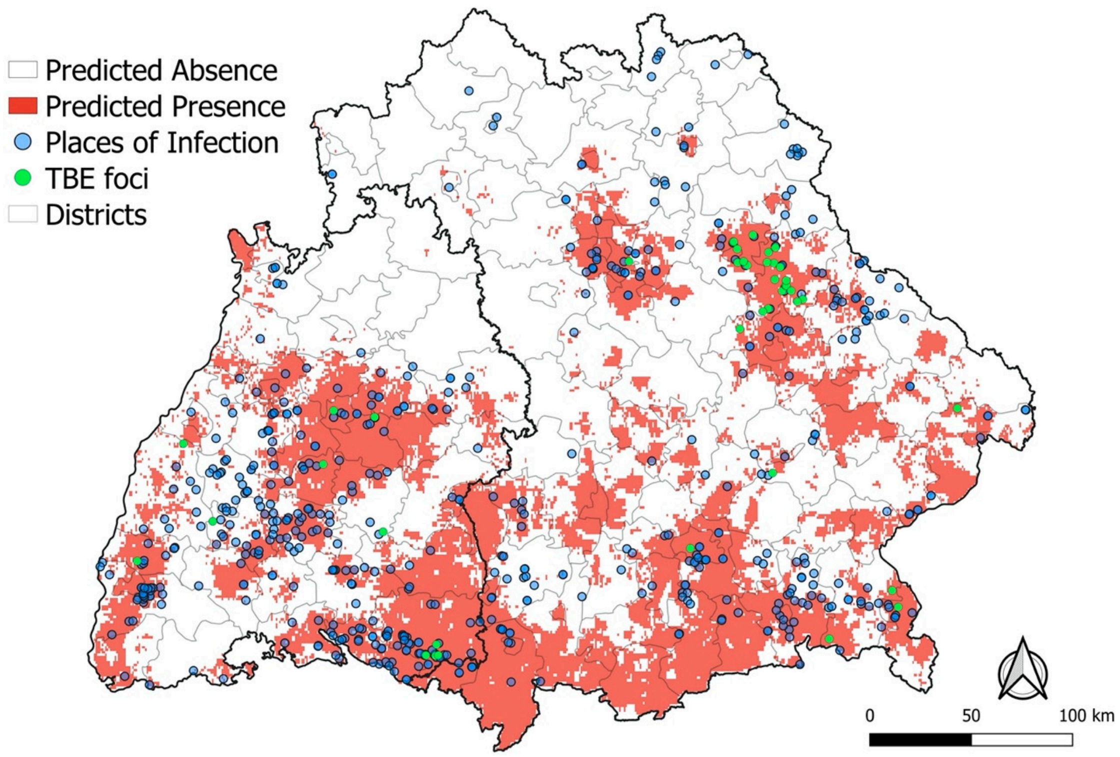

Figure S4. Binary map based on the optimised probability threshold maximising the sum of sensitivity and specificity of the ecological niche model predictions, superimposed with places of TBE infection and TBE foci; the cut-off probability threshold for place-of-TBE-infection presence is at 0.28 with sensitivity 69% and specificity 63%.

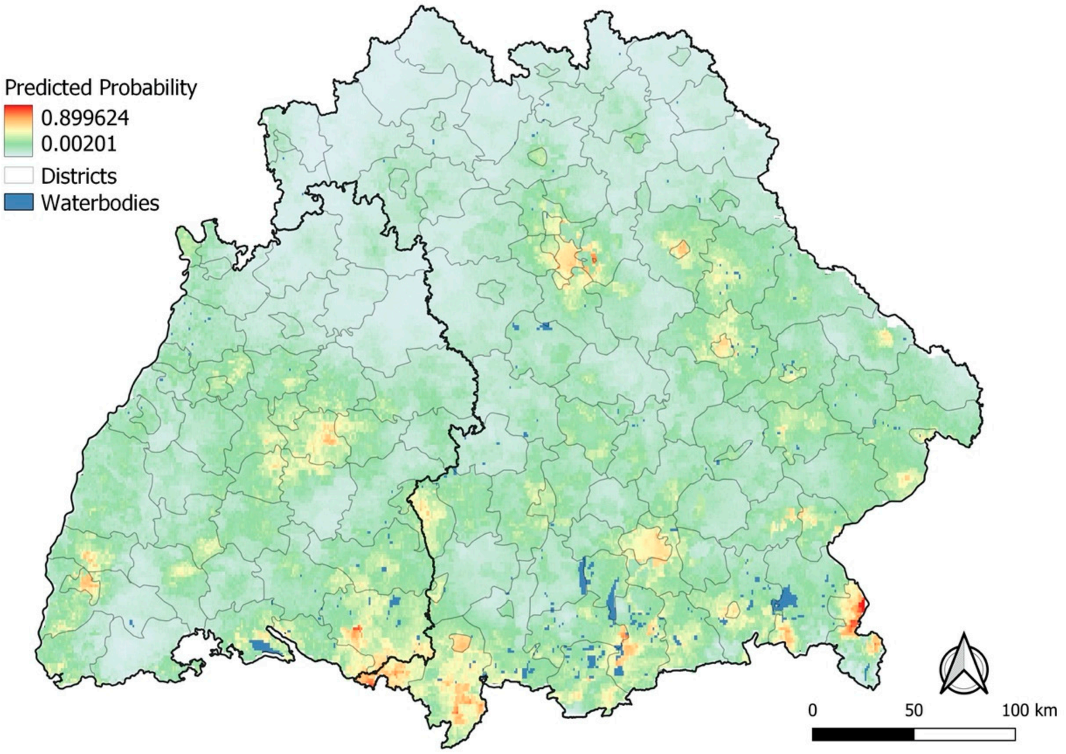

Figure S5. Predictive map from sensitivity analysis with presence data limited to only TBE foci and places of TBE infection where a tick bite occurred (59% of all places of TBE infection).

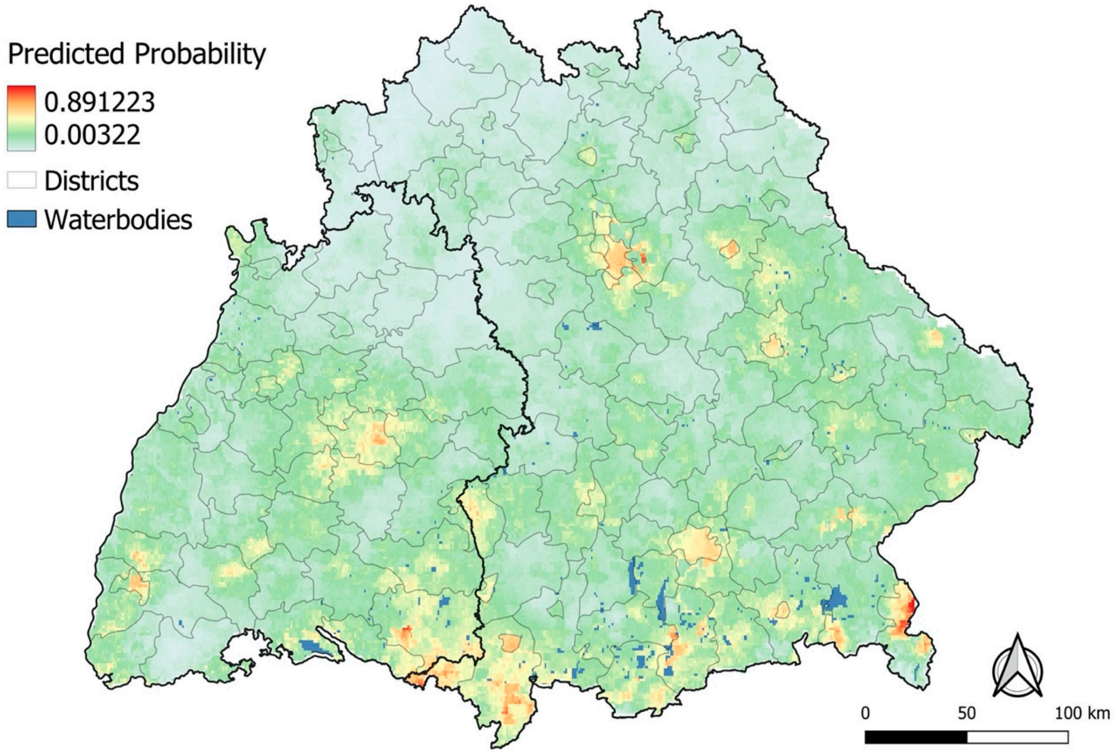

Figure S6. Predictive map from sensitivity analysis with presence data limited to only TBE foci and places of TBE infection smaller than 1 km² (66% of all places of TBE infection).

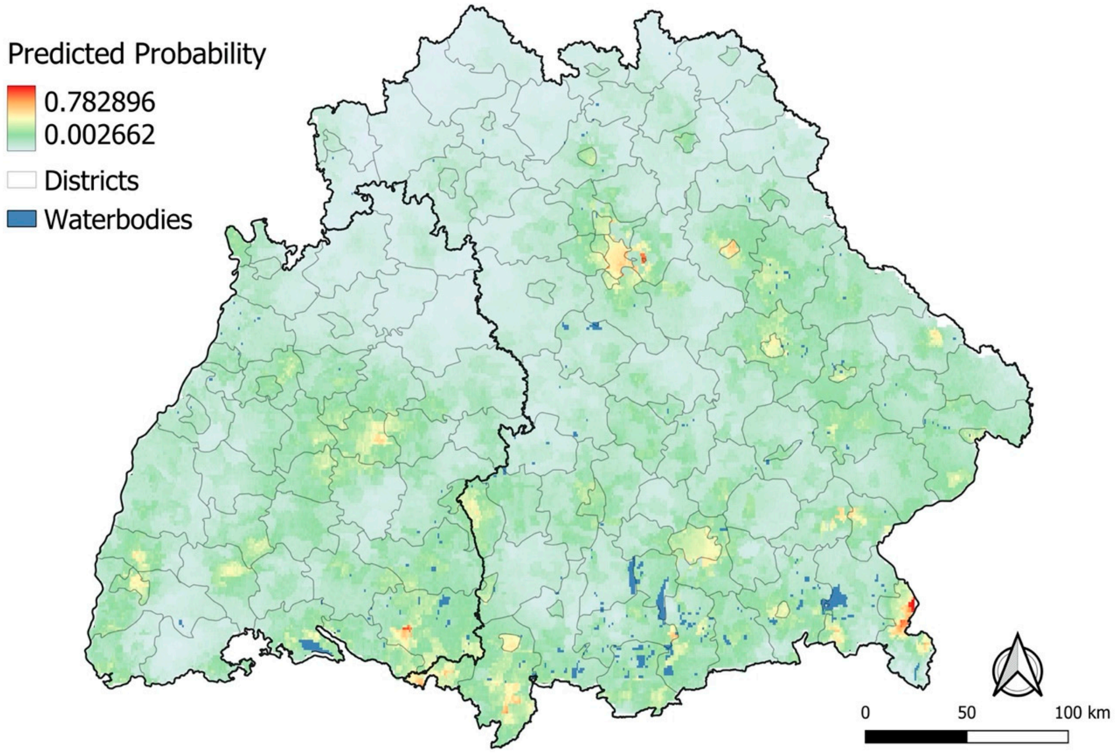

Figure S7. Predictive map from sensitivity analysis with presence data limited to only TBE foci and places of TBE infection that have been reported as standalone places by a case (weight = 1) (33% of all places of TBE infection).

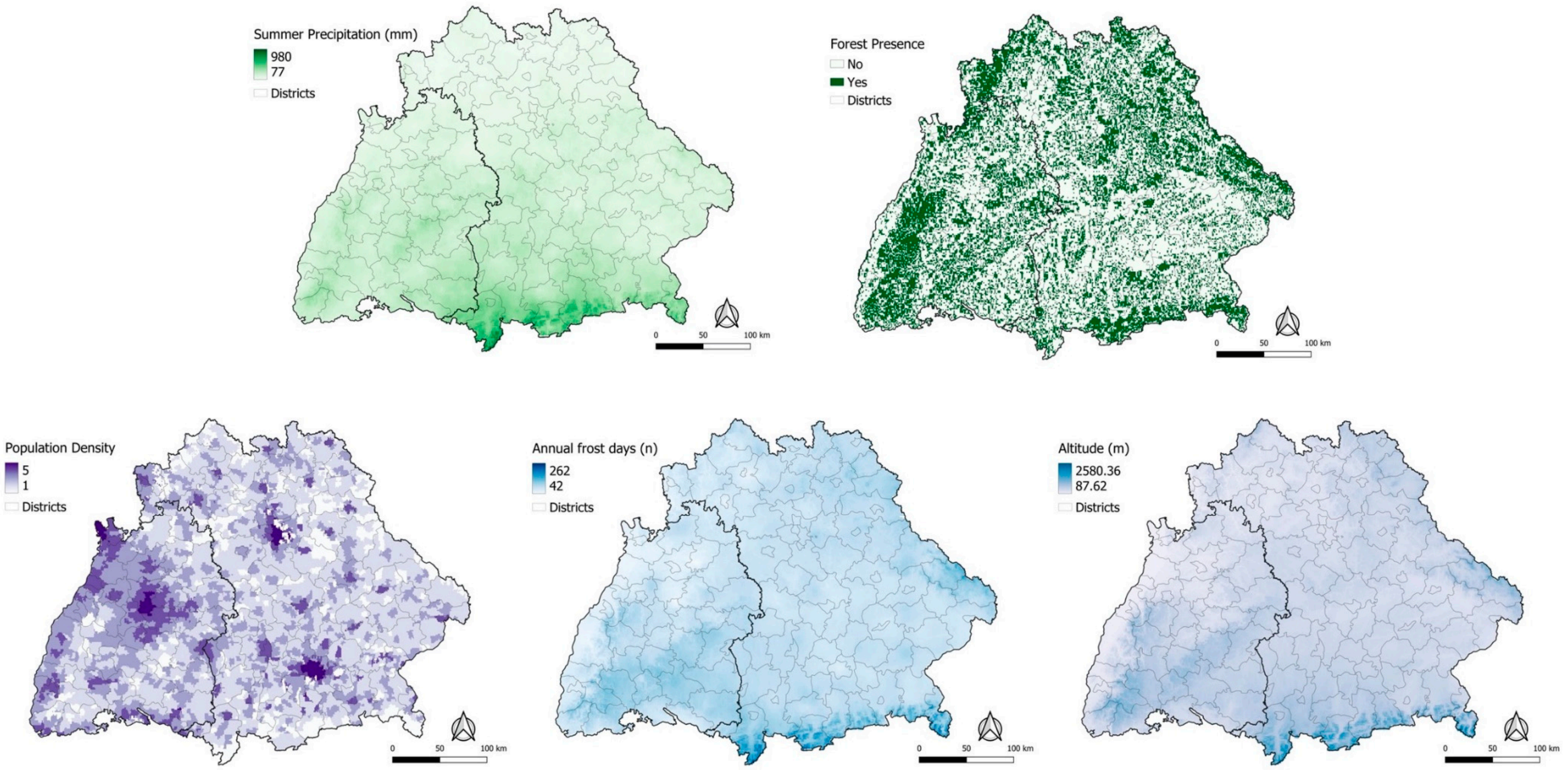

Figure S8. Raster files of summer precipitation (mm), forest presence (yes/no), population density (level 1–5), annual frost days (n), and altitude (m); Levels for population density are measured in inhabitants (n) per km^2: 1 = 0–50/km^2, 2 = 50–150/km^2, 3 = 150–500/km^2, 4 = 500–2000/km^2, 5 = 2000–4790/km^2.

Table S1. Descriptive statistics of ecological covariates at places of TBE infection, natural TBE foci, and comparator places after VIF selection.

	Mean	Standard Deviation	Minimum	Maximum
**Mean summer temperature (°C)**				
Places of infection	18.7	1.1	14.8	21.5
TBE foci	19.1	0.6	17.7	20.3
Comparator places	18.7	1.0	13.5	21.5
**Number of hot days (> 30 °C) per year (n)**				
Places of infection	15.3	6.4	3.0	37.0
TBE foci	19.0	4.7	10.7	29.0
Comparator places	15.4	5.3	2.1	33.7
**Number of frost days (< 0 °C) per year (n)**				
Places of infection	90.7	14.1	53.8	131.3
TBE foci	92.2	10.1	63.9	108.3
Comparator places	91.2	15.3	48.0	164.8
**Mean summer precipitation height (mm)***				
Places of infection	264.2	108.6	77.0	761.4
TBE foci	247.4	80.6	180.8	484.3
Comparator places	236.9	85.7	107.5	667.1
**Number of snow cover days (>= 1 cm) per year (n)**				
Places of infection	26.8	16.3	0.0	94.5
TBE foci	21.3	10.2	0.0	46.7
Comparator places	27.3	18.0	0.0	140.8
**Tick density (n/100m^2)**				
Places of infection	125.3	68.1	10.4	656.0
TBE foci	143.7	96.2	7.1	421.4
Comparator places	149.4	72.2	1.1	660.0

Table S2. Raw odds ratios (OR) and 95% confidence intervals (95% CI) for places of TBE infection presence for all model covariates.

**Covariate**	**OR**	**95% CI**
Population Density	1.76	(1.55, 2.01)
Forest presence	1.21	(0.98, 1.59)
Vaccination coverage	1.05	(1.03, 1.08)
Summer temperature	1.05	(1.02, 1.08)
Frost days	1.04	(1.03, 1.06)
Summer precipitation	1.01	(1.01, 1.01)
Tick density	1.00	(1.00, 1.00)
Snow cover days	0.98	(0.96, 0.99)

* These values should be interpreted with caution since the size of the units differed substantially between some covariates and may therefore impact comparability of the odds ratios.
